# Comparison of the Saponins in Three Processed American Ginseng Products by Ultra-High Performance Liquid Chromatography-Quadrupole Orbitrap Tandem Mass Spectrometry and Multivariate Statistical Analysis

**DOI:** 10.1155/2022/6721937

**Published:** 2022-04-26

**Authors:** Na Guo, Yuxin Bai, Xin Huang, Xiaokang Liu, Guangzhi Cai, Shuying Liu, Yunlong Guo, Jiyu Gong

**Affiliations:** ^1^Jilin Ginseng Academy, Changchun University of Chinese Medicine, Changchun 130117, China; ^2^School of Pharmaceutical Sciences, Changchun University of Chinese Medicine, Changchun 130117, China

## Abstract

A method with ultrahigh performance liquid chromatography Quadrupole-Orbitrap tandem mass spectrometry (UHPLC-Q-Orbitrap-MS/MS) was applied for the quality evaluation of different processing and drying of American ginseng, including natural drying (ND), steam drying (SD), and vacuum freeze-drying (VFD). A total of 51 saponins were successfully identified in three processed products. Three processed American ginseng products were well-differentiated in orthogonal partial least-squares discriminant analysis (OPLS-DA). The S-plot also identified the marker compounds in each product, while the major ginsenosides of ND (malonyl (M)-Rd, M-Rb_1_, Rg_1_), SD (20 (S)-Rg_3_, 20 (S)-Rg_2_), and VFD (M-Rd, M-Rb_1_) were found. The results indicate that the method by vacuum freeze-drying can retain the content of rare ginsenosides and malonyl-ginsenosides. The marker compounds selected will benefit the holistic evaluation of related American ginseng products.

## 1. Introduction

American ginseng (*Panax quinquefolium* L.) is well-known for replenishing Qi in Chinese medicine [1, 2]. American ginseng contains several bioactive compounds, including polysaccharides, saponins, amino acids, volatile oil, and mineral elements, while ginsenoside is one of the important active ingredients [3–9].

Ginseng has three popular processes, including natural air drying, steaming drying, and vacuum freeze-drying, respectively [10–17]. The drying process affects the quality of ginseng products and changes the content of ginsenosides. The essence of the change in ginsenoside content is the transformation of ginsenosides during the drying process. Thermal processing (natural air drying and steaming drying) converts saponins of larger molecular weight into saponins of smaller molecular weight. Ginsenoside M-Rb_1_, M-Rb_2_, M-Rc, Re, Rg_1_, Rb_1_, Ginsenoside Rb_2_, Rc, Rd, Re, Rg_1_, and Ginsenoside Rd, Rk_1_, Rg_5_, Rg_3_ are the major ginsenosides of white ginseng, red ginseng, and black ginseng, respectively [15, 18, 19]. Nonthermal processing (vacuum freeze-drying) can keep the shape and color of ginseng consistent with its fresh state, containing more natural active ingredients. The characteristic components in vacuum freeze-drying ginseng were M-Re, M-Rb_1_, M-Rc, M-Rb_1_ isomer, M-Rb_2_, M-Rb_3_ and M-Rd isomer [11].

The LC-MS technique combines the high separation ability for complex samples with the high selectivity of high-resolution mass spectrometry and the ability to provide information (molecular weight and structural) and is widely used to control quality standards for traditional Chinese medicine [20–22]. The composition of Chinese herbal medicines has been rapidly analyzed by UHPLC-Q-Orbitrap-MS/MS and the changes in their chemical composition before and after processing as an effective tool for identifying active ingredients with improved sensitivity and accuracy. The fragment information of tandem mass spectrometry can be used to identify the structure of compounds [23–25].

Multivariate analysis methods were applied to analyze whether differences existed between test samples and to determine which compounds were altered for quality evaluation of herbal medicines of different origins, parts, and processing methods [11, 23, 24, 26, 27]. Recent studies demonstrated that the UPLC-QTOF/MS is an optimal application for holistic evaluation of ginseng [18, 28]. Principal component analysis (PCA) is distinguished from the compounds of different drying processes of Houttuyniae Herba [29].

The common processed products of American ginseng are dried American ginseng [30–32]. It is necessary to control the drying process in the processing of American ginseng [33–35]. In recent years, steamed American ginseng and vacuum freeze-dried American ginseng have appeared in the functional food market. However, the systematic comparison of ginsenoside conversions of natural drying (ND), steam drying (SD), and vacuum freeze-drying (VFD) has not yet been studied.

In this paper, UHPLC-Q-Orbitrap-MS/MS analysis combined with multivariate analysis approach was applied to evaluate the composition of ND, SD, and VFD. This study aims to explore the trends of transformation of ginsenosides and characterize and quantify the chemical ingredients in three processed American ginseng products to standardize the processing procedures reasonably.

## 2. Materials and Methods

### 2.1. Chemicals and Reagents

The chemicals and reagents used were as follows: methanol, acetonitrile, and formic acid (HPLC grade, Fisher Scientific); reference ginsenosides Rh_1_, Rg_5_, Rk_1_, Rg_3_, F_2_, Re, Rg_1_, Rb_1_, Rg_2_, Rc, Rd; and pseudoginsenoside F_11_ (Shanghai Yuanye Biotechnology Co., Ltd.) were obtained. The purity of the standards was no less than 98%.

### 2.2. Processed American Ginseng Samples

**Figure 1:**
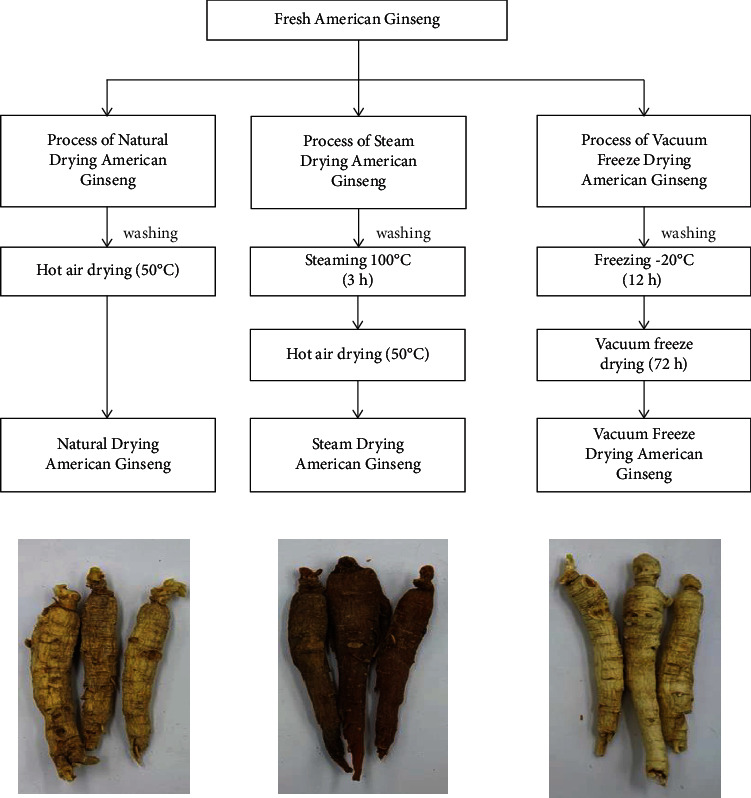
The scheme of American ginseng products: natural drying (ND), steam drying (SD), and vacuum freeze-drying (VFD).

The samples of fresh American ginseng were first cleaned and then processed by the experiment (steaming drying, natural air drying, and vacuum freeze-drying). The ND samples were produced by drying at 50°C. The SD samples were produced in a ginseng steaming cabinet at 100°C for 3 h and allowed to dry in an oven at 50°C. The VFD samples were produced by first prefreezing at −20°C for 12 h and then placing them in a vacuum freeze-dryer (Ningbo Xinzhi Biotechnology Co., Ltd., Zhejiang, China) to freeze-dry for 72 h, as shown in Figure 1.

### 2.3. Preparation of Standard and Sample Solutions

A series of reference mixtures with ginsenosides Rh_1_, Rg_5_, Rk_1_, Rg_3_, F_2_, Re, Rg_1_, Rb_1_, Rg_2_, Rc, Rd, and pseudoginsenoside F_11_ were dissolved in 70% methanol-water to a final concentration of 0.2 mg/mL. Each of the stock solutions was combined to obtain final concentrations.

A fine powder (0.1 g) was ultrasonically extracted with 70% methanol-water (5 mL) for 45 min. The extraction was filtered through a 0.22 *μ*m syringe filter before analysis [36].

### 2.4. Instrument and Condition

A UPLC system (Ultimate 3000) was used for separations. A Supelco C_18_ column (3.0 × 50 mm, 2.7 *μ*m) was used at 35°C for separation and eluted by the mobile phases (solvent A and B) were acetonitrile and water containing 0.1% formic acid, separately. The gradient elution program with a 0.5 mL/min flow rate was as follows: 15%-15% A (0–2 min); 15–30% A (2–15 min); 30–95% A (15–25 min); 95–15% A (25–27 min); and 15–15% A (27–35 min). The injection volume was 10 *µ*L.

A Q-Orbitrap-MS/MS via an ESI source in the negative ion mode. For the ESI source, sheath gas flow of 35 Arb, aux gas flow of 10 Arb, sweep gas flow of 1 Arb, capillary voltage of −3.5 kV, and capillary temperature of 350°C. A full MS data were scanned with m/*z* 150–2000 Da, 70,000 resolution, automatic gain control (AGC) target, 1 × 10^6^, and maximum injection time (IT), 100 ms. The dd-MS^2^ was scanned with 17,000 resolution, AGC target, 1 × 10^5^, IT, 50 ms, loop count 5, isolation window 4.0 m/*z*, and NCE, 25–55. Then, a Full-MS/dd-MS^2^ mode was used for analysis.

### 2.5. Data Analysis

The data were processed by SIEVE 2.1 (Thermo Fisher, San Jose, CA, USA). Then, the datasets were multivariate analyzed by SIMCA-P software 11.5 (Umetrics, Umea, Sweden). The components with VIP values larger than 1 and *p* < 0.05 were selected as analytical markers in the OPLS-DA model and the *t*-test by SPSS 19.0 (Chicago, IL, USA), separately.

## 3. Result and Discussion

### 3.1. Analysis of Three Processed American Ginseng Products by UHPLC-Q-Orbitrap-MS/MS

**Figure 2:**
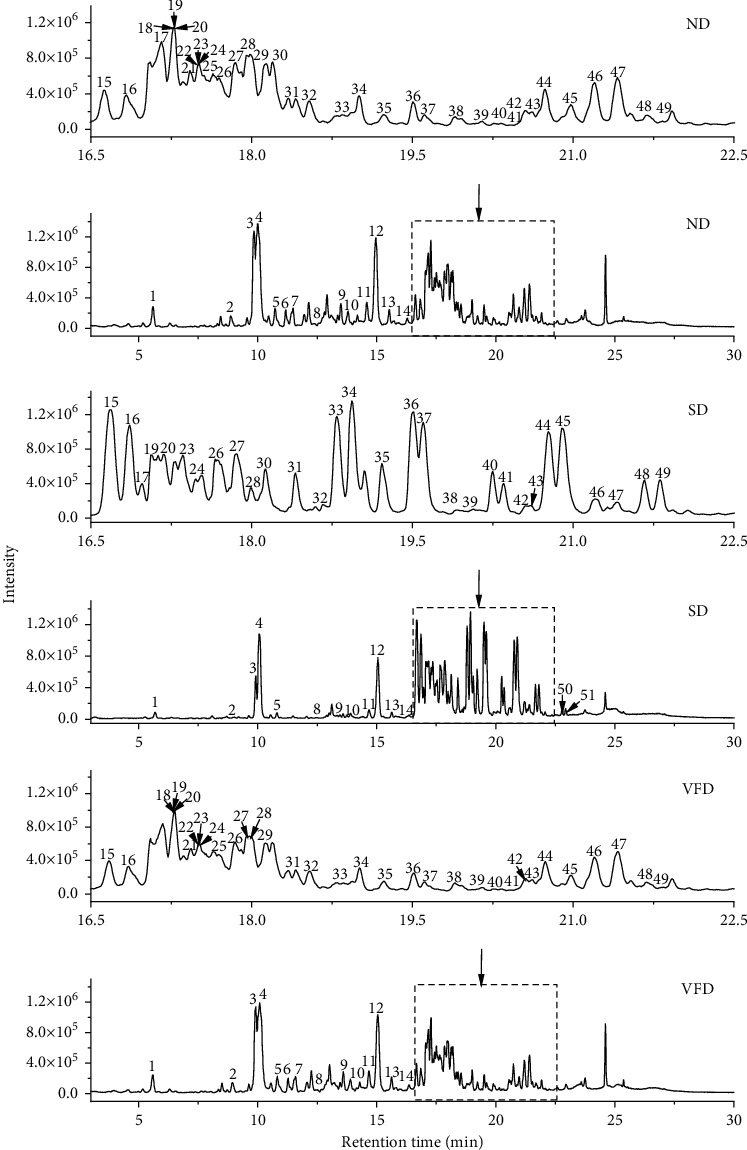
Base peak chromatogram of American ginseng of natural drying (ND), steam drying (SD), and vacuum freeze-drying (VFD) in the negative ion mode.

The method of UHPLC-Q-Orbitrap-MS/MS was applied for determination [23, 37, 38]. The identification of the extracts of ND, SD, and VFD samples in the negative ion mode is shown in Figure 2. The compounds were separated distinctly in 30 min by UPLC. The distinct samples of several peaks demonstrated the different intensities at 15–25 min. The ratio of Rg_1_ and Re was changed with steaming treatment, and the content of Rg_1_ in SD samples was significantly decreased at 10 min. Meanwhile, the content of minor ginsenosides was increased at 18–22 min. A slice of studies performed showed that the major ginsenosides (Re and Rg_1_) were converted to minor ginsenosides (Rh_4_, Rk_1_, Rk_3_, 20 (S)-Rg_3_, Rg_5_, and 20 (R)-Rg_3_) by heat treatment [39]. The content of malonyl-ginsenosides in VFD samples was higher than in SD samples. These results demonstrated the change in ginsenoside composition of ND, SD, and VFD samples.

The Q-Orbitrap-MS accurately measured the compounds' mass values [24]. Meanwhile, the fragmentation pattern of standards was compared to identified ginsenosides from three processed American ginseng products.

**Figure 3:**
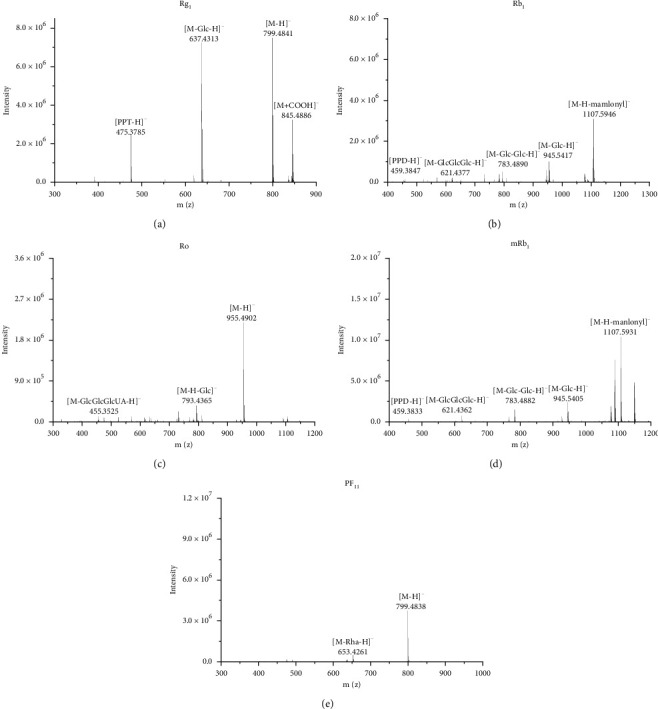
The MS/MS spectrum of ginsenosides in the negative ion mode: (a) Rg1; (b) Rb1; (c) Ro; (d) M-Rb1; (e) PF11.

The full-scan MS was used to confirm the molecular weight. Ginsenosides were easily ionized into [M − H]^−^ and [M + HCOO]^−^ ions in the negative ion mode. All molecular ions were unambiguously identified within the mass accuracy of 10 ppm. The different tandem MS spectra of the various aglycone types are shown in Figure 3. In Figure 3(a), fragment ion at m/*z* 475 indicated the successive losses of two glucose residues. In Figure 3(b), the successive losses of four glucose residues were observed from fragment ion at m/*z* 459. The losses of two glucose residues and a *β*-d-glucuronic acid (176 Da) were observed from fragment ion at m/*z* 455 in Figure 3(c). In Figure 3(d), the loss of a malonyl residue was observed from malonyl-ginsenoside at m/*z* 1107, and the fragment ion at m/*z* 459 was similarly produced by successive loss of four glucose residues. In Figure 3(e), the loss of a rhamnose residue (146 Da) was observed from fragment ion at m/*z* 653. In total, 51 ginsenosides were identified from ND, SD, and VFD by comparing standards and literature records of the tandem MS spectra (Table 1).


(1) $$\begin{array}{c} \text{a}{\left[\text{M}+\text{COOH}\right]}^{-};\\ \text{b}{\left[\text{M}-\text{H}\right]}^{-}. \end{array}$$

### 3.2. Multivariate Statistical Analysis

The statistical methods were applied to display the differences in ginsenosides intuitively. After data preprocessing of the ND, SD, and VFD samples, the dataset was conducted to discover the marker compound by multivariate statistical analysis.

**Figure 4:**
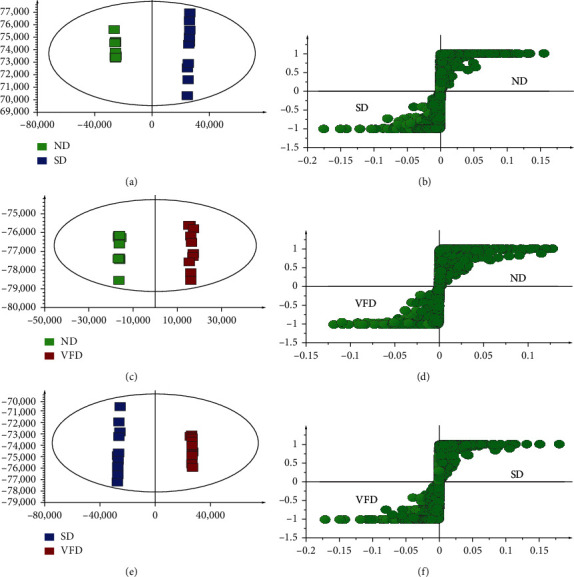
The OPLS-DA/S-plot of ND vs. SD, ND vs. VFD, and SD vs. VFD.

The distinct ginsenoside composition of samples from ND, SD, and VFD was characterized in detail. The OPLS-DA model was effectively used to observe three processed American ginseng products (Figure 4). An excellent prediction ability was considered to the parameters of SD vs. ND (R2 Y = 0.865, Q2 = 0.999), VFD vs. ND (R2 Y = 0.822, Q2 = 0.998), and VFD vs. SD (R2 Y = 0.848, Q2 = 1). The score plot was used to discriminate between the groups of two selected samples (ND vs. SD, ND vs. VFD, and SD vs. VFD). The samples of ND were compared to the other samples by OPLS-DA, separately. By comparing the S-plot of ND and SD, the components observed from the lower left quadrant and the upper right quadrant were elevated in SD and ND, respectively (Figure 4(b)). The marker components were elevated in SD (20(S)-Rg3, 20(S)-Rg2, and an unknown compound) and ND (malonyl (M)-Rb1, M-Rd). Four components (20(S)-Rg3, 20(S)-Rg2, M-Rb1, and M-Rd) were identified as marker compounds in SD and ND. By comparing the S-plot of ND and VFD, it was performed that four unknown compounds and two compounds (M-Rb1, Rg1) were elevated in VFD and ND, respectively (Figure 4(d)). The ginsenosides of M-Rb1 and Rg1 were identified as marker compounds in ND and VFD. By comparing SD and VFD, it was performed that two malonyl-ginsenosides (M-Rd, M-Rb1) and three compounds (20(S)-Rg3, 20(S)-Rg2, and an unknown compound) were elevated in VFD and SD, respectively (Figure 4(f)). Four ginsenosides of M-Rd, M-Rb1, 20(S)-Rg3, and 20(S)-Rg2 were identified as marker compounds in VFD and SD. The repeated emergence of marker compounds was in different groups, including the SD group (20(S)-Rg3 and 20(S)-Rg2) and the ND group (M-Rb1). Meanwhile, the marker compounds could display various pharmacological activities such as antitumor (20(S)-Rg3) [40], against cardiocerebrovascular diseases (20(S)-Rg2) and affect the central nervous system (M-Rb1) [41, 42].

**Figure 5:**
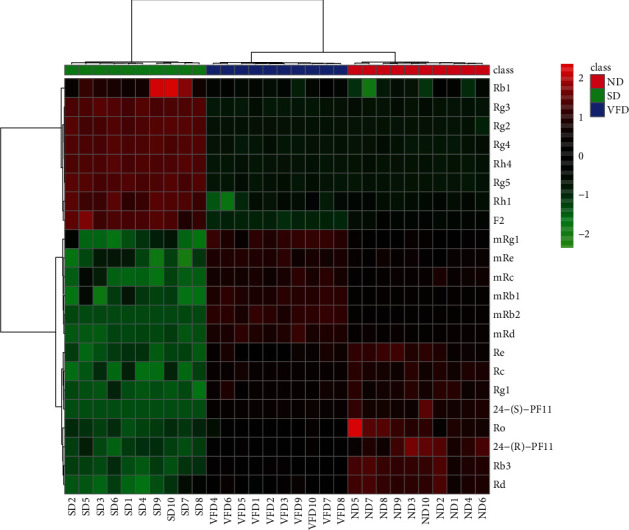
The heat map visualizes the intensities of the 22 ginsenosides datasets from ND, SD, and VFD (*n* = 10).

The VIP value was used to select the marker compounds [29]. The features with VIP values larger than 1 were highlighted as marker candidates. The compounds with the statistically significant difference (*p* < 0.05) were screened for marker compounds by ANOVA. To visualize the tendency of marker compounds, the intensities of 22 marker compounds were constructed as a heat map. The composition change was shown in different colors by the ND group, the SD group, and the VFD group (Figure 5). Between the SD and ND groups, the contents of ginsenoside-Rb_1_, -Rh_1_, -Rg_3_, -Rg_2_, -Rg_4_, -Rh_4_, –F_2_, and -Rg_5_ in SD samples were increased, and the contents of malonyl-ginsenoside-Rg_1_, -Re, -Rc, -Rb_1_, -Rb_2_, -Rd, and ginsenoside-Re, -Rc, -Rg_1_, -Rb_3_, -Rd, -Ro, 20-(S)-PF_11_, and 20-(R)-PF_11_ in ND samples were significantly higher. Comparing the groups of ND and VFD, the contents of malonyl-ginsenoside-Rg_1_, -Re, -Rc, -Rb_1_, -Rb_2_, and -Rd in the VFD group were significantly increased. Meanwhile, the contents of ginsenoside-Re, -Rc, -Rg_1_, -Rb_3_, -Ro, -Rd, 20-(S)-PF_11_, and 20-(R)-PF_11_ in the ND samples were significantly increased. The malonyl residue of malonyl-ginsenoside in ND and VFD was well preserved without heating treatment. Similar to ginseng, it was observed that the content of malonyl-ginsenosides in American ginseng was increased by a vacuum freeze-drying approach [11]. The thermal sensitivities of malonyl-ginsenosides were less affected by the vacuum freeze-drying technology.

The malonyl-ginsenoside of chemical transformation is related to heat and pressure treatment [24]. Meanwhile, the alteration of three processed American ginseng products is possibly related to the degree of enzymatic activity, moisture, and microstructure [43]. The content of moisture can result in a rapid transformation by promoting corresponding microbiological growth, enzymatic and nonenzymatic reactions in American ginseng. The active compounds are cleaved by enzymes with steam and high temperatures [9, 44]. As a result, the content of ginsenoside Rb_1_ in SD samples was higher than in VFD and ND samples. The content of ginsenoside Rg_1_ in ND and VFD samples was higher than in SD samples; it probably converted into 20(S)-Rg_3_, 20(R)-Rg_3_, Rk_1_, and Rg_5_ by heat treatment [39]. Malonyl-ginsenosides in VFD and ND samples were higher than in SD samples, and they were probably converted into malonic acid through decarboxylation by heat treatment. In summary, these factors all lead to the transformation of ginsenosides in the three processed products. The composition and content of components in various processed products can affect their pharmacological activity, and it is necessary to elucidate the change of ginsenosides.

### 3.3. Quantification of Ginsenosides from Three Processed American Ginseng Products

The 12 compounds' EIC was identified by the reference compounds in Figure S1 and Table S1 (Supplementary material). The standard curve was used to determine the actual amount of the three processed American ginseng products in Figure S2 and Table S2 (Supplementary material). The quantification was based on 12 ginsenosides for products (*n* = 3) and the amount of ginsenosides was calculated in Table S3 (Supplementary material).

Previously, Huang et al. has reported the 59 ginsenosides of protopanaxadiol, the concentrations of ginsenosides Rk_1_, Rg_5_, Rh_1_, 20(R)-ginsenoside Rg_2_, and 20(R)-ginsenoside Rg_3_ are highest in red American Ginseng, these results have been matched to ours [10]. For ginsenosides analysis, the content of ginsenosides Rg_1_, Re, Rb_1_, Rc, Rd, and 24(R)-pseudo-ginsenoside F_11_ is the highest in ND. As our results, the concentrations of ginsenosides Rg_1_, Re, Rb_1_, Rc, Rd, and 24(R)-pseudo-ginsenoside F_11_ are highest in ND, and this is matched to another paper [18]. Ginsenoside Rh_1_ is not detected in ND and VFD.

## 4. Conclusions

A successful method was performed for the chemical components of three processed American ginseng products using UHPLC-Q-Orbitrap-MS/MS. The 5 ginsenosides as characteristic marker compounds could be applied to elucidate the composition of ND, SD, and VFD samples by multivariate statistical analysis. The results will be useful to visualize the tendency of marker compounds in manufacturing. Furthermore, this study effectively provided a means for assessing and controlling different processed American ginseng products.

## Figures and Tables

**Table 1:** Compounds identified from American ginseng by natural drying, steam drying, and vacuum freeze-drying.

No.	Rt (min)	Identification	Formula	Detected mass (Da)	Mass error (ppm)
1	5.67	Vina-ginsenoside R_4_	C_48_H_82_O_19_	1007.5457^a^	2.5
2	9.04	Notoginsenoside R_1_	C_47_H_80_O_18_	977.5346^a^	1.9
3	10.03	Ginsenoside Rg_1_	C_42_H_72_O_14_	845.4914^a^	1.2
4	10.41	Ginsenoside Re	C_48_H_82_O_18_	991.5504^a^	2.1
5	10.73	24 (S)-pseudo-ginsenoside F_11_	C_42_H_72_O_14_	845.4922^a^	2.1
6	11.45	Malonyl-ginsenoside Rg_1_	C_45_H_74_O_17_	885.4867^b^	1.6
7	11.51	Malonyl-ginsenoside Re	C_51_H_84_O_21_	1031.5457^b^	2.4
8	12.69	20 (S)-notoginsenoside R_2_	C_41_H_70_O_13_	815.4819^a^	2.6
9	13.47	Ginsenoside F_5_	C_41_H_70_O_13_	815.4814^a^	2.0
10	13.85	Acetyl-Rg_1_	C_44_H_74_O_15_	887.5024^a^	1.6
11	14.80	Pseudo-RT_2_	C_41_H_70_O_14_	785.4661^b^	4.1
12	15.26	24 (R)-pseudo-ginsenoside F_11_	C_42_H_72_O_14_	845.4918^a^	1.6
13	15.72	Notoginsenoside R_4_/Ginsenoside Ra_3_	C_59_H_100_O_27_	1285.6464^a^	1.6
14	16.46	Ginsenoside Rh_1_	C_36_H_62_O_9_	683.4391^a^	2.2
15	16.61	20 (S)-ginsenoside Rg_2_	C_42_H_72_O_13_	829.4971^a^	1.9
16	16.89	20 (R)-ginsenoside Rg_2_	C_42_H_72_O_13_	829.4974^a^	2.3
17	17.01	Ginsenoside Rb_1_	C_54_H_92_O_23_	1153.603^a^	1.6
18	17.06	Malonyl-ginsenoside Rb_1_/Isomer	C_57_H_94_O_26_	1193.5983^b^	1.8
19	17.13	Ginsenoside Rc	C_53_H_90_O_22_	1123.5920^a^	1.2
20	17.20	Ginsenoside Ro	C_48_H_76_O_19_	955.4933^b^	2.6
21	17.27	Malonyl-ginsenoside Rc	C_56_H_92_O_25_	1163.5883^b^	2.4
22	17.29	Malonyl-ginsenoside Rb_2_	C_56_H_92_O_25_	1163.5892^b^	3.2
23	17.36	Ginsenoside Rb_2_	C_53_H_90_O_22_	1123.5923^b^	1.5
24	17.59	Ginsenoside Rb_3_	C_53_H_90_O_22_	1123.5924^a^	1.6
25	17.66	Malonyl-ginsenoside Rb_3_	C_56_H_92_O_25_	1163.5878^b^	2.0
26	17.73	Pseudo-RT_1_	C_47_H_74_O_18_	971.4800^a^	5.9
27	17.84	Ginsenoside Rd	C_48_H_82_O_18_	991.5506^a^	2.3
28	18.07	Chikusetsusaponin IVa	C_42_H_66_O_14_	793.4396^b^	2.0
29	18.10	Malonyl-ginsenoside Rd	C_51_H_84_O_21_	1031.5458^b^	2.5
30	18.14	Gypenoside XVII	C_48_H_82_O_18_	991.5502^a^	1.9
31	18.40	Pseudo-RC_1_	C_50_H_48_O_19_	1033.5521^a^	6.6
32	18.63	Quinquefolium III	C_50_H_48_O_19_	1033.5529^a^	5.8
33	18.80	Ginsenoside Rg_6_	C_42_H_70_O_12_	811.4810^a^	4.8
34	18.90	Ginsenoside Rg_4_	C_42_H_70_O_12_	811.4807^a^	5.2
35	19.24	Ginsenoside F_2_	C_42_H_72_O_13_	829.4971^a^	1.9
36	19.58	20 (S)-ginsenoside Rg_3_	C_42_H_72_O_13_	829.4976^a^	2.5
37	19.63	Ginsenoside Rk_3_	C_36_H_60_O_8_	665.4231^a^	5.9
38	19.79	Ginsenoside Rh_4_	C_36_H_60_O_8_	665.4233^a^	5.6
39	20.01	Zingibroside R_1_	C_42_H_66_O_14_	839.4382^a^	5.1
40	20.27	20 (R)-ginsenoside Rg_3_	C_42_H_72_O_13_	829.4982^a^	3.3
41	20.34	20 (S)-ginsenoside Rs_3_	C_44_H_74_O_14_	871.5015^a^	5.3
42	20.60	Calenduloside E	C_36_H_56_O_9_	677.3869^a^	5.5
43	20.68	20 (R)-ginsenoside Rs_3_	C_44_H_74_O_14_	871.5014^a^	5.3
44	20.88	Ginsenoside Rk_1_	C_42_H_70_O_12_	811.4859^a^	1.2
45	20.92	Ginsenoside Rg_5_	C_42_H_70_O_12_	811.4868^a^	2.3
46	21.19	20 (S)-ginsenoside Rh_2_	C_36_H_62_O_8_	667.4391^a^	5.4
47	21.42	20 (R)-ginsenoside Rh_2_	C_36_H_62_O_8_	667.4393^a^	5.1
48	21.62	Ginsenoside Rs_5_	C_44_H_72_O_13_	853.4913^a^	4.7
49	21.85	Ginsenoside Rs_4_	C_44_H_72_O_13_	853.4913^a^	4.7
50	23.18	Ginsenoside Rk_2_	C_36_H_60_O_7_	649.4276^a^	6.9
51	23.33	Ginsenoside Rh_3_	C_36_H_60_O_7_	649.4294^a^	4.2

## Data Availability

The data used to support findings of this study are included within the article.

## Supplementary Materials

Supplementary Materials Supplementary Materials. SFigure 1: exacted icon chromatogram of target compounds in ginsenosides extracts. SFigure 2: twelve ginsenosides from standard curves and three processed American ginseng products. STable 1: mass spectrometry parameters of target compounds. STable 2: linear data of the target compounds. STable 3: quantification of 12 ginsenosides from three processed American ginseng products.
