# Supplementary material for: Comparison of the Saponins in Three Processed American Ginseng Products by Ultra-High Performance Liquid Chromatography-Quadrupole Orbitrap Tandem Mass Spectrometry and Multivariate Statistical Analysis
Source: Int J Anal Chem. 2022 Apr 26;2022:6721937. doi: 10.1155/2022/6721937 (PMC9064508; doi:10.1155/2022/6721937)
Supplement: Supplementary Materials — SFigure 1: exacted icon chromatogram of target compounds in ginsenosides extracts. SFigure 2: twelve ginsenosides from standard curves and three processed American ginseng products. STable 1: mass spectrometry parameters of target compounds. STable 2: linear data of the target compounds. STable 3: quantification of 12 ginsenosides from three processed American ginseng products. [file 6721937.f1.docx]

***Supporting Information***

**Comparison on the Saponins in Three Processed American Ginseng Products by Ultra-high Performance Liquid Chromatography-Quadrupole Orbitrap Tandem Mass Spectrometry and Multivariate Statistical Analysis**

Na Guo,^#1^ Yuxin Bai,^#2^ Xin Huang,^1^ Xiaokang Liu,^2^ Guangzhi Cai,^2^ Shuying Liu,^1^ Yunlong Guo,*^1^ and Jiyu Gong*^2^

^1^Jilin Ginseng Academy, Changchun University of Chinese Medicine, Changchun 130117, China

^2^School of Pharmaceutical Sciences, Changchun University of Chinese Medicine, Changchun 130117, China

Correspondence should be addressed to Yunlong Guo; guoyl02@ccucm.edu.cn and Jiyu Gong; gjy0431@126.com

Na Guo and Yuxin Bai contributed equally to this work.

**T_ABLE_ S1**: Mass spectrometry parameters of target compounds

| Compounds | RT (min) | Selected ion | Formula | Theoretical  m/z | Experimental  m/z | Mass error (ppm) |
| --- | --- | --- | --- | --- | --- | --- |
| Ginsenoside Rg_1_ | 10.03 | [M+COOH]- | C_43_H_73_O_16_ | 845.4904 | 845.4866 | 4.5 |
| Ginsenoside Re | 10.41 | [M+COOH]- | C_49_H_83_O_20_ | 991.5483 | 991.5448 | 3.8 |
| 24(R)-Pseudo-ginsenoside F_11_ | 15.26 | [M+COOH]- | C_43_H_73_O_16_ | 845.4904 | 845.4870 | 4.0 |
| Ginsenoside Rh_1_ | 16.46 | [M+COOH]- | C_37_H_63_O_11_ | 683.4376 | 683.4349 | 4.0 |
| 20(R)-Ginsenoside Rg_2_ | 16.89 | [M+COOH]- | C_43_H_73_O_15_ | 829.4955 | 829.4926 | 3.5 |
| Ginsenoside Rb_1_ | 17.01 | [M+COOH]- | C_55_H_93_O_25_ | 1153.6011 | 1153.5962 | 4.2 |
| Ginsenoside Rc | 17.13 | [M+COOH]- | C_54_H_91_O_24_ | 1123.5906 | 1123.5863 | 3.8 |
| Ginsenoside Rd | 17.84 | [M+COOH]- | C_49_H_83_O_20_ | 991.5483 | 991.5448 | 3.5 |
| Ginsenoside F_2_ | 19.24 | [M+COOH]- | C_43_H_73_O_15_ | 829.4955 | 829.4922 | 4.0 |
| 20(R)-Ginsenoside Rg_3_ | 20.27 | [M+COOH]- | C_43_H_73_O_15_ | 829.4955 | 829.4932 | 2.8 |
| Ginsenoside Rk_1_ | 20.88 | [M+COOH]- | C_43_H_71_O_14_ | 811.4849 | 811.4824 | 3.1 |
| Ginsenoside Rg_5_ | 20.92 | [M+COOH]- | C_43_H_71_O_14_ | 811.4849 | 8111.4821 | 3.4 |

**T_ABLE_ S2**: Linear data of the target compounds

| Compounds | Calibration curve | r | Linear range/(μg/mL) |
| --- | --- | --- | --- |
| Ginsenoside Rg_1_ | y=3678452649.4498x+4412303.9500 | 0.9960 | 0.15~78.6 |
| Ginsenoside Re | y=2060641578.4743x+2523270.9932 | 0.9984 | 0.21~107.8 |
| Ginsenoside Rb_1_ | y=426859526.3838x+403770.3937 | 0.9997 | 0.21~107.8 |
| Ginsenoside Rd | y=1220974854.1217x+723682.8863 | 0.9995 | 0.15~74.2 |
| Ginsenoside Rc | y=197614216.9344x+576524.1567 | 0.9968 | 0.50~255 |
| 24(R)-Pseudo-ginsenoside F_11_ | y=1787646970.7908x+1448452.6099 | 0.9976 | 0.15~76.4 |
| 20(R)-Ginsenoside Rg_3_ | y=245739554.0633x+740634.9251 | 0.9955 | 0.50~253.8 |
| 20(R)-Ginsenoside Rg_2_ | y=196268171.6428x-335.1380 | 0.9980 | 0.54~277.5 |
| Ginsenoside Rh_1_ | y=601785218.2571x+349336.2967 | 0.9987 | 0.32~163.5 |
| Ginsenoside Rk_1_ | y=67374043.6056x+322979.0760 | 0.9966 | 7.47~382.5 |
| Ginsenoside Rg_5_ | y=200078956.5445x+18250.7878 | 0.9951 | 4.50~230 |
| Ginsenoside F_2_ | y=313043891.0033x+686992.9114 | 0.9982 | 0.54~275.8 |

**T_ABLE_ S3**: Quantification of 12 ginsenosides from three processed American ginseng products. Values are expressed as % (n= 3).

| No. | RT (min) | Ginsenosides | ND | SD | VFD |
| --- | --- | --- | --- | --- | --- |
| 1 | 10.03 | Ginsenoside Rg_1_ | 0.0686±0.0055 | 0.0583±0.0051 | 0.0658±0.0046 |
| 2 | 10.41 | Ginsenoside Re | 0.0671±0.0046 | 0.0584±0.0078 | 0.0655±0.0047 |
| 3 | 15.26 | 24(R)-Pseudo-ginsenoside F_11_ | 0.0502±0.0053 | 0.0374±0.0052 | 0.0474±0.0052 |
| 4 | 16.46 | Ginsenoside Rh_1_ | Not Detected | 0.0175±0.0004 | Not Detected |
| 5 | 16.89 | 20(R)-Ginsenoside Rg_2_ | 0.0057±0.0014 | 0.1750±0.0067 | 0.0063±0.0012 |
| 6 | 17.01 | Ginsenoside Rb_1_ | 0.0693±0.0049 | 0.0594±0.0049 | 0.0680±0.0046 |
| 7 | 17.13 | Ginsenoside Rc | 0.0542±0.0047 | 0.0315±0.0046 | 0.0446±0.0048 |
| 8 | 17.84 | Ginsenoside Rd | 0.0378±0.0052 | 0.0316±0.0043 | 0.0357±0.0047 |
| 9 | 19.24 | Ginsenoside F_2_ | 0.0039±0.0053 | 0.0580±0.0006 | 0.0021±0.0038 |
| 10 | 20.27 | 20(R)-Ginsenoside Rg_3_ | 0.0097±0.0008 | 0.1110±0.0060 | 0.0038±0.0004 |
| 11 | 20.88 | Ginsenoside Rk_1_ | 0.0023±0.0016 | 0.1970±0.0084 | Not Detected |
| 12 | 20.92 | Ginsenoside Rg_5_ | 0.0020±0.0005 | 0.0824±0.0054 | 0.0018±0.0006 |

**F_IGURE_ S1**: Exacted icon chromatogram of target compounds in ginsenosides extracts (A) and reference solution (B).

((1) Ginsenoside Rg_1_, (2) Ginsenoside Re, (3) 24(R)-Pseudo-ginsenoside F_11_, (4) Ginsenoside Rh_1_, (5) 20(R)-Ginsenoside Rg_2_, (6) Ginsenoside Rb_1_, (7) Ginsenoside Rc, (8) Ginsenoside Rd, (9) Ginsenoside F_2_, (10) 20(R)-Ginsenoside Rg_3_, (11) Ginsenoside Rk_1_ and (12) Ginsenoside Rg_5_)

**F_IGURE_ S2**: Twelve ginsenosides from standard curves and three processed American ginseng products.
